# Combined Spatial and Dosimetric Recurrence Pattern Analysis in Head and Neck Squamous Cell Carcinoma Following Postoperative (Chemo)radiotherapy

**DOI:** 10.1186/s13014-025-02641-8

**Published:** 2025-04-23

**Authors:** Philipp Schröter, Hoi Hin Lau, Florian Stritzke, Henrik Franke, Katharina Weusthof, Sebastian Regnery, Lukas Bauer, Maximilian Deng, Katharina Dvornikovich, Anna Hofmann, Lars Wessel, Karl Semmelmayer, Julius Moratin, Oliver Ristow, Jürgen Hoffmann, Peter Plinkert, Gerhard Dyckhoff, Jürgen Debus, Thomas Held

**Affiliations:** 1 Department of Radiation Oncology, Heidelberg University Hospital, Heidelberg University, Heidelberg, Germany; 2 Heidelberg Institute of Radiation Oncology (HIRO), Heidelberg, Germany; 3 National Center for Tumor diseases (NCT), Heidelberg, Germany; 4 Clinical Cooperation Unit Radiation Oncology, German Cancer Research Center (DKFZ), Heidelberg, Germany; 5 Heidelberg Ion Beam Therapy Center (HIT), Heidelberg, Germany; 6 German Cancer Consortium (DKTK), partner site Heidelberg, German Cancer Research Center (DKFZ), Heidelberg, Germany; 7 Department of Oral and Cranio-Maxillofacial Surgery, Heidelberg University Hospital, Heidelberg University, Heidelberg, Germany; 8 Department of Otorhinolaryngology, Head and Neck Surgery, Heidelberg University Hospital, Heidelberg University, Heidelberg, Germany

**Keywords:** Recurrence patterns, Postoperative radiation therapy, Oral cavity cancer, Head and neck cancer, Local control, Dosimetric analysis, Radiotherapy de-escalation

## Abstract

**Background:**

Advancements in nodal staging for head and neck squamous cell carcinoma (HNSCC) have prompted radiotherapy de-escalation trials to reduce irradiation of electively treated neck regions, with the goal of improving treatment tolerability. While volumetric de-escalation has shown promise in definitive radiotherapy of HNSCC, limited data exist regarding its safety in the postoperative treatment setting. This study aimed to assess dose-level-specific locoregional recurrence patterns following standard postoperative (chemo)radiotherapy in a mixed HNSCC cohort to inform risk-adaptive radiotherapy strategies.

**Materials and methods:**

We retrospectively reviewed 203 HNSCC patients (75% HPV-negative, 25% HPV-positive) treated with curative intent postoperative (chemo)radiotherapy from 2017 to 2021. Recurrence imaging was co-registered with planning CT, and recurrent tumor volumes were dosimetrically compared to the target volume dose and spatially analyzed using a center-of-mass-based approach. We classified five recurrence types: A (central high-dose), B (peripheral high-dose), C (central intermediate- or low-dose), D (peripheral intermediate- or low-dose), and E (extraneous dose).

**Results:**

With a median follow-up of 39.7 months, the three-year local, regional, and distant control of HPV-negative HNSCC were 84%, 87%, and 87%, respectively. Of 56 recurrences, 17 were local, 13 regional, 3 locoregional, 9 combined local/regional with concomitant distant failure, and 14 distant only. Of 40 analyzed recurrences, we identified 47.5% as type A/B, 5% as type C/D intermediate-dose, and 20% as type E, half of which were secondary cancers. Among the 27.5% (11/40) type C/D low-dose recurrences in the elective target volume, 15% (6/40) were true nodal failures, resulting in an overall elective neck failure rate of 3% (6/203).

**Conclusion:**

The predominance of high-dose recurrences suggests that biological tumor resistance is a key driver of treatment failure, highlighting the necessity to refine postoperative risk stratification and integrate tumor biology into dose escalation decisions. The low incidence of isolated nodal recurrences in electively treated neck regions supports the feasibility of volumetric de-escalation of postoperative radiotherapy. This approach might not only be feasible for HPV-associated oropharyngeal cancers but also for HPV-negative tumors, provided that accurate nodal staging has been conducted.

**Supplementary Information:**

The online version contains supplementary material available at 10.1186/s13014-025-02641-8.

## Background

While the fundamental approach to multimodal treatment for locoregionally advanced head and neck squamous cell carcinoma (HNSCC) has remained consistent over the past decades, considerable technical advancements have been made in therapy components. Intensity-modulated radiotherapy (IMRT) has enabled increasingly conformal, multi-dose-leveled postoperative radiotherapy (PORT) for HNSCC, allowing for better normal tissue sparing. However, these complex IMRT plans carry a heightened risk of missing the tumor geographically and require precise treatment delivery. Therefore, refined analyses of recurrence patterns are essential for quality control in radiation therapy and for guiding the evolution of HNSCC target delineation.

As target volume definition and dose delivery in IMRT have technically advanced, so too have methods for analyzing recurrence patterns, providing a more accurate reflection of the complexity in multi-volume and multi-dose IMRT plans [1–3].

Conventional recurrence reporting based solely on anatomical or field references (in-field vs. marginal vs. out-of-field) was sufficient for conventional three-dimensional conformal radiotherapy (3D-CRT) with homogeneous and sizable RT volumes. However, it might not be suitable for more complex IMRT plans. A more recent combined spatial and dosimetric method integrates the point-of-origin approach with dosimetry of recurrence volumes, offering a nuanced, etiology-indicative classification of failure types [2, 4]. Generally, correlating tumor recurrence volumes with initial clinicopathological features, anatomical locations, and treatment dosimetry can suggest the etiology of recurrences. In-field recurrences typically indicate tumor resistance, while marginal or out-of-field recurrences may reflect suboptimal target volume delineation or errors in treatment delivery.

Target volume delineation for PORT in HNSCC is less standardized than for definitive RT and recurrence patterns appear to be more diverse. This might be attributable to the introduction of new tissue planes facilitating abnormal patterns of tumor spread. Therefore, in the past, a comprehensive postoperative treatment approach of the primary tumor site and, in many cases, the bilateral cervical lymphatic drainage was preferred. However, as imaging techniques and the accuracy of nodal-staging have improved, prospective clinical trials are increasingly exploring volumetrically de-intensified treatment plans, aiming to enhance treatment tolerability in the postoperative setting [5–7]. Reducing irradiation volumes conventionally advised to receive elective irradiation doses, such as neck areas or neck sites, that have been surgically staged negative, is one such strategy.

Evaluating dose-level specific recurrence patterns in a representative HNSCC cohort treated with postoperative IMRT (PO-IMRT) could inform future approaches of volumetrically de-intensified target delineation.

We therefore performed a detailed recurrence pattern analysis of 203 HNSCC patients treated with PO-IMRT at the Heidelberg University Hospital, Department of Radiation Oncology, between 2017 and 2021, applying a combined spatial and dosimetric analysis method.

## Methods

### Patient selection

This was a retrospective single-center study. The integrated Heidelberg Institute for Radiation Oncology (HIRO) database was used to identify patients with diagnosis of squamous cell carcinoma of the oral cavity (OCSCC), oropharynx (OPSCC), hypopharynx (HPSCC) or larynx (LSCC), treated with curative intent PO-IMRT at the Heidelberg University Hospital, Department of Radiation Oncology from 2017 to 2021. Patients with prior surgery or RT for HNSCC, documented progression of disease during PO-IMRT or discontinuation of PO-IMRT with > 5 fractions missing were excluded.

### Clinical data collection

Diagnostic contrast-enhanced computed tomography (CT) or magnetic resonance imaging (MRI) documenting first evidence of local and/or regional recurrence and/or distant metastases (rCT/rMRI) was identified. Recurrence was confirmed by pathology specimen (i.e. surgical biopsy) in most cases, and in all cases by interdisciplinary tumor board case review. Patient, disease and treatment characteristics were recorded from chart review.

### Treatment planning and delivery

All patients were immobilized using a thermoplastic head mask. Intraoral stents were used at the discretion of the treating radiation oncologist. CT scans with 3 mm–1 mm slice thickness were used for treatment planning. Contrast-enhanced CT and/or T1-weighted MRI and surgery/pathology reports were used for target volume delineation. Treatment planning was conducted using RayStation (RayStation 11B, RaySearch Laboratories, Stockholm, Sweden). According to standard procedures at our institution and in concordance with international guidelines [8–14] organs at risk were delineated. Three clinical target volumes (CTV1-3), corresponding to high, intermediate and low-risk areas were typically defined as recommended by The Danish Head and Neck Cancer Group (DHANCA) Guidelines 2013 and 2020 [13]. A planning target volume (PTV) margin of 3 mm was added to CTV1-3. Doses prescribed to these 3 targets were 66, 60, and 54–57 Gy, respectively, delivered in 30 fractions. Occasionally a high-risk volume was identified that received higher dose (66–72.6 Gy). Radiation treatment was performed as IMRT in 200 patients and as combined RT (IMRT and carbon ion or proton boost) in 3 patients, using the active raster scanning method. For patients with recurrent disease at the start of RT, doses could be individualized. Daily image guidance was performed by cone-beam CT for IMRT and by orthogonal x-rays in case of carbon ion or proton boost irradiation, with position correction.

### Follow-up

Imaging follow-up, including contrast-enhanced CT or MRI scans of the head and neck were scheduled 6 to 8 weeks after treatment and then every 3 months within the first two years, every 6 months within the third year, and every 12 months within year four to five. A CT thorax scan and abdominal sonography was performed once per year. Symptoms and toxicities were recorded non-standardized by a radiation oncologist at each follow-up visit according to the Common Terminology Criteria for Adverse Events (CTCAE Version 4.03 and 5.0). Patients presented regularly to an otorhinolaryngologist or maxillofacial surgeon for clinical examination.

### Image registration

**Fig. 1:**
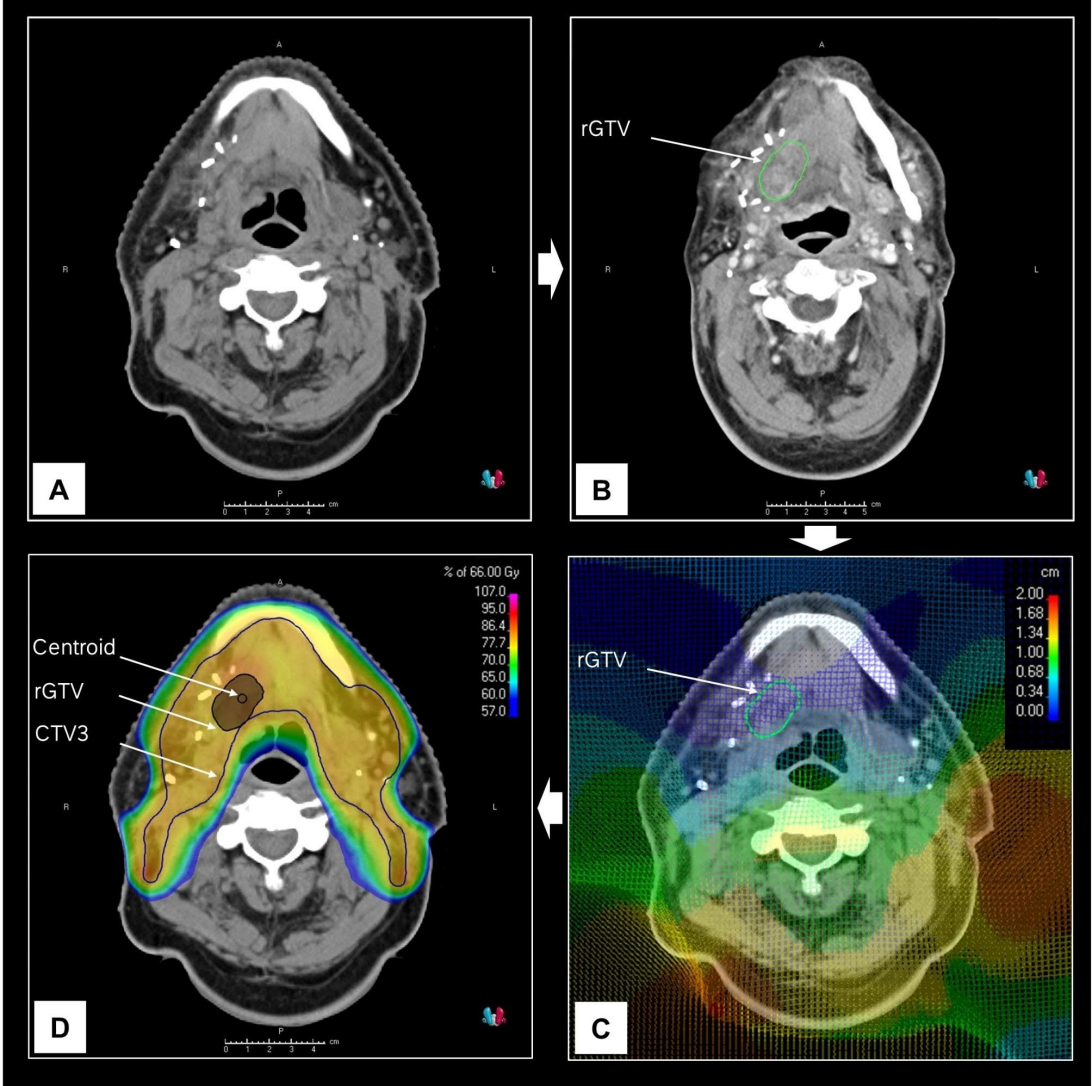
Workflow of recurrence gross tumor volume (rGTV) delineation and deformable image registration (DIR). (**A**) planning CT (pCT) and radiotherapy plan were retrieved. (**B**) recurrence CT or MRI (rCT/rMRI) documenting recurrence was retrieved for rGTV delineation. (**C**) rCT/rMRI was co-registered with pCT. (**D**) rGTV on the rCT/rMRI was deformed to co-registered pCT and the centroid was calculated

Using clinical information and radiologic imaging, recurrent gross tumor volumes (rGTV) were delineated on the rCT or rMRI. Original planning CT (pCT) and irradiation plans were restored. A rigid frame-of-reference registration was first performed between the pCT and the rCT/rMRI, followed by deformable image registration (DIR) with the pCT as the reference. An anatomically constrained deformation algorithm based on image intensity and anatomical information was used for DIR. The deformed rGTV was then propagated to the pCT, and a 4 mm diameter centroid was delineated using a 2 mm margin around the central voxel of the rGTV (Fig. 1).

### Pattern of recurrence classification

Recurrences were classified into five different types [2] (Table 1). The centroid of the deformed rGTV determined the target volume of origin. The dose to 95% of the rGTV (rD95%) was calculated and compared relative to the prescribed dose of the target volume of origin. A recurrence was classified as a secondary cancer if the rGTV occurred at a site unrelated to the initial primary cancer and was confirmed as such by review through the interdisciplinary tumor board. In the case of multiple rGTV, the higher classification type was considered dominant.

**Table 1:** Recurrence classification

Recurrence type	Recurrence volume characteristics
A (central high dose)	centroid rGTV located in PTV60-72.6 Gy, rD95% ≥ 95% of prescribed dose.
B (peripheral high dose)	centroid of rGTV located in PTV60-72.6 Gy, rD95% < 95% of prescribed dose
C (central intermediate/low dose)	centroid of rGTV located in PTV57/PTV54Gy, rD95% ≥ 95% of prescribed dose
D (peripheral intermediate/low dose)	centroid of rGTV located in PTV57/PTV54Gy, rD95% < 95% of prescribed dose
E extraneous dose	centroid of rGTV outside any PTV

rGTV = recurrent gross tumor volume, rD95% = dose to 95% of the recurrent gross tumor volume

### Event definitions

Local treatment failure was defined as recurrence at the primary tumor site, regional failure as recurrence in cervical lymph nodes, and distant failure as recurrence in organs outside the head and neck.

### Statistical analysis

Statistical analysis was performed using GraphPad Prism (Version 10.1.2) and R (Version 4.4.1). Local, regional, and distant treatment failure were estimated separately using cumulative incidence function. The time to event was measured from the start of PO-IMRT. Deaths without the event of interest, lost to follow up or treatment failure patterns different from the event of interest were considered competing risk events. The time to event following recurrence was measured from the date of diagnosis of recurrence. Postrecurrence overall survival (OS) was estimated using the Kaplan-Meier method. The median follow-up for OS was calculated using the inverse Kaplan-Meier method.

## Results

### Patient, disease, treatment characteristics and outcomes (entire population)

**Fig. 2:**
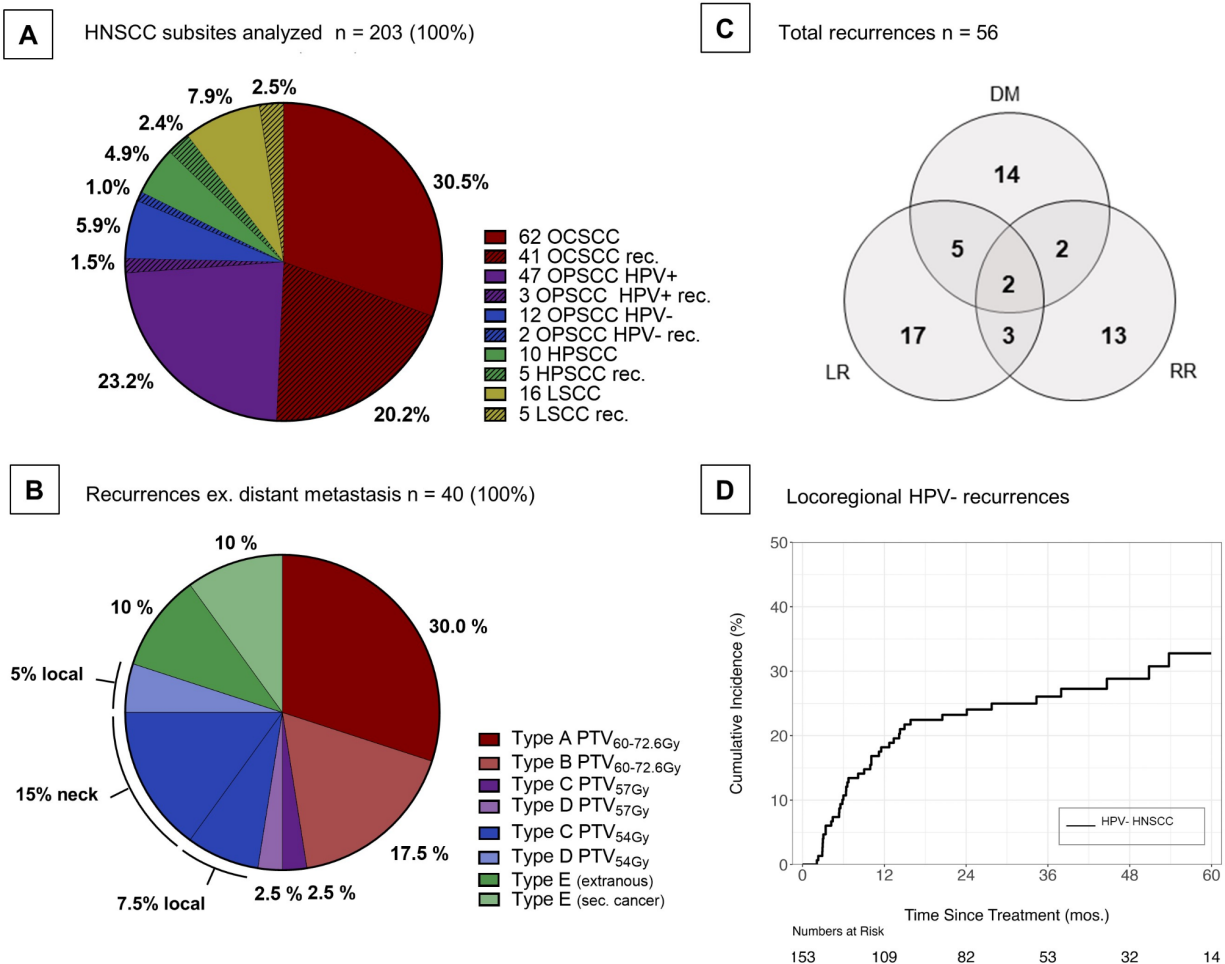
HNSCC subsites, distribution of recurrence types and cumulative incidence of locoregional recurrence for HPV- HNSCC. (**A**) Pie chart depicting proportions of HNSCC subsites of the entire dataset, including the relative fraction of recurrences. (**B**) Pie chart depicting the distribution of predominant typology of recurrence, including the location of type C/D low-dose recurrences. (**C**) Venn diagram depicting pattern of local/regional recurrence and distant metastasis (**D**) Cumulative incidence of locoregional recurrences for HPV- HNSCC, taking competing risk into account. HNSCC = head and neck squamous cell carcinoma, OCSCC = oral cavity squamous cell carcinoma, OPSCC = oropharyngeal squamous cell carcinoma, HPSCC = hypopharyngeal squamous cell carcinoma, LSCC = laryngeal squamous cell carcinoma, HPV = human papillomavirus, DM = distant metastasis, LR = local recurrence, RR = regional recurrence, ex. = excluding, mos.= months

In total 206 patients receiving curative intent PO-IMRT between 2017 and 2021, without prior HNSCC treatment, were identified. Three patients were excluded due to disease progression during PO-IMRT or treatment discontinuation with > 5 fractions not applied. The median follow-up was 39.7 months. Excluding patients with HPV-positive OPSCC (50/203) the 3-year locoregional control rate in the HPV-negative mixed-case cohort was 74% (Fig. 2D), 3-year local, regional and distant control were 84%, 87% and 87% respectively (Supplementary Fig. 1A-C). Of the 203 patients 64% received bilateral, 33% received ipsilateral and 3% received no neck dissection, while 77% received bilateral, 17% received unilateral and 6% received no neck irradiation (Table 2). Of patients who received unilateral or no neck irradiation, none had recurrence in the unirradiated neck.

**Table 2:** Treatment characteristics

**Recurrence population, excluding DM only**	***n*** **=** **40**	**(%)**
IMRT Dose and Fractionation		
Median Dose (range), Gy	66	(60-72.6)
Median Fractionation (range)	30	(30–33)
Boost to High-Risk Volumes		
66–72.6 Gy	29	(72.5)
Concurrent Systemic Therapy		
Cisplatin	13	(32.5)
Carboplatin	3	(7)
Cetuximab	1	(2.5)
None	23	(56)
Due to comorbidities	9	(39)
Time to Initiation of postoperative (C)RT		
≤ 6 weeks	16	(39)
> 6 weeks	24	(61)
Total Treatment Package Time		
Median (IQR), days	92	(80–104)
< 77 days	3	(7.5)
77–85 days	12	(30.0)
86–100 days	12	(30.0)
> 100 days	13	(32.5)
**Recurrence population**,** including DM only**	***n*** **=** **56**	**(%)**
Salvage therapy		
Local therapy	23	(41)
Resection of isolated pulmonary metastases	3	
Re-Resection local/regional	19	
Re-RT	1	
Systemic therapy	21	(38)
None/BSC	8	(14)
Unknown	4	(7)
Neck dissection		
Bilateral	41	(73.0)
Unilateral	13	(23.0)
None	2	(4.0)
Neck irradiation		
Bilateral	50	(89.0)
Unilateral	3	(5.5)
None	3	(5.5)
**Entire population**	***n*** **=** **203**	**(%)**
Neck dissection		
Bilateral	130	(64)
Ipsilateral	67	(33)
None	6	(3)
Neck irradiation		
Bilateral	156	(77)
Ipsilateral	34	(17)
None	13	(6)

DM = distant metastasis, (C)RT = (chemo)radiotherapy, IQR = interquartile range, BSC = Best Supportive Care

### Patient, disease, treatment characteristics and outcomes (recurrence population)

Of the 203 patients analyzed, 56 developed recurrence. Median time to recurrence was 7.5 months (range 2–73 months). Seventeen patients (30.4%) had local recurrence, 13 (23.2%) had regional recurrence, 3 (5.4%) had combined locoregional recurrence and 9 (16.0%) experienced local, regional or locoregional recurrence with concomitant distant metastasis, while 14 (25.0%) developed sole distant metastasis (Fig. 2C). Patients with distant metastases only and 2 patients without imaging documenting recurrence were excluded, leaving 40 patients for recurrence pattern analysis (Table 3; Fig. 2B, Supplemental Table 1). Median total treatment package time (TTP) for the 40 recurrence patients was 92 days (interquartile range: 80–104 days) vs. 87 days (interquartile range: 79–96 days) in patients without recurrence. In all 13/40 (33%) recurrence patients with TTP ≥ 100 days (a negative prognosticator [15–17]), the reason for prolonged TTP was a delay in PO-IMRT commencement. Specific causes were revision-surgery (3/13), prolonged wound healing (3/13), re-resection due to R1 margin (2/13), patient-related reasons (2/13), unspecified (2/13) or other medical events (1/13). Postrecurrence outcomes were analyzed for the largest recurrence site subset, which were OCSCC patients. The Kaplan-Meier estimate for median OS was 12.7 months following local, regional or locoregional recurrence vs. 3.2 months following distant metastasis (Supplementary Fig. 1D).

**Table 3:** Patient and disease characteristics

Recurrence population, excluding DM only	*n* = 40	(%)
Age		
Median (range), years	66.0	(23–86)
Gender		
Female	16	(40.0)
Male	24	(60.0)
Tumor site		
Oral Cavity	33	(82.5)
Oral Tongue	11	(27.5)
Buccal Mucosa	1	(2.5)
Floor of Mouth	5	(12.5)
Upper/Lower Alveolar Ridge and Gingiva	16	(40.0)
Oropharynx (p16-)	1	(2.5)
Hypopharynx	2	(5.0)
Larynx	4	(10)
Histologic differentiation		
Poor	7	(17.5)
Moderate	32	(80.0)
Well	0	
p16+	1	(2.5)
Pathological T stage		
T1	3	(7.5)
T2	4	(10.0)
T3	10	(25.0)
T4a	22	(55.0)
T4b	1	(2.5)
Pathological N stage		
No dissection	1	(2.5)
N0	12	(30.0)
N1	5	(12,5)
N2a	1	(2,5)
N2b	5	(12,5)
N2c	1	(2,5)
N3a	0	
N3b	15	(37.5)
Overall stage		
Stage I	0	
Stage II	1	(2.5)
Stage III	5	(12.5)
Stage IVa	19	(47.5)
Stage IVb	15	(37.5)
Primary surgery margin status		
Negative (> 5 mm)	10	(25.0)
Close (1–5 mm)	17	(42.5)
Positive	11	(27.5)
Unspecified	2	(5.0)
Depth of invasion		
≤ 5 mm	4	(10.0)
> 5 mm ≤ 10 mm	8	(20.0)
> 10 mm	17	(42.5)
Unspecified	11	(27.5)
Perineural invasion		
Yes	18	(45.0)
No	17	(42.5)
Unspecified	5	(12.5)
Lymphovascular invasion		
Yes	23	(57.5)
No	17	(42.5)
Extracapsular extension		
Yes	15	(55.5)
No	12	(44.5)

### Pattern of recurrences

Twelve patients (30.0%) were classified type A (PTV60-72.6 Gy), 7 (17.5%) type B (PTV60-72.6 Gy), 1 (2.5%) type C intermediate-dose (PTV57Gy), 1 (2.5%) type D intermediate-dose (PTV57Gy), 9 (22.5%) type C low-dose (PTV54Gy), 2 (5%) type D low-dose (PTV54Gy) and 8 (20%) experienced type E failures, of which 4 (10%) were secondary cancers (Fig. 2B).

Of the 11 patients with type C/D low-dose recurrence, 5 (12.5%) occurred locally in proximity to the primary tumor and 6 (15%) occurred in elective lower neck volumes.

### Cases of local recurrences

Of 16 analyzable patients with local recurrence only and 5 patients with local recurrence and concomitant distant failure, 11 (52%) had high-dose failures (7 type A and 4 type B). Of the 10 (48%) non high-dose failures 3 were type C low-dose, 2 were type D low-dose and 5 were type E. Focusing on non-central high-dose failures: of the 4 type B failures 3 were due to outgrowth of the recurrence volume from the PTV1. The fourth type B case denotes a patient with a long history of leukemia; therefore, it was agreed on a small volume PO-IMRT only whereby CTV2 and CTV3 were omitted deliberately, however the recurrence occurred directly on the PTV1 margin.

The 3 patients with type C low-dose failure had resected carcinomas of the right lateral border of the tongue with flap reconstruction. Two showed recurrences at the flap interface, extending into the right floor of mouth representing outgrowth from higher to lower dose regions. The third patient presented with a recurrence on the left lateral border of the tongue after 72.5 months considered as a secondary cancer.

**Fig. 3:**
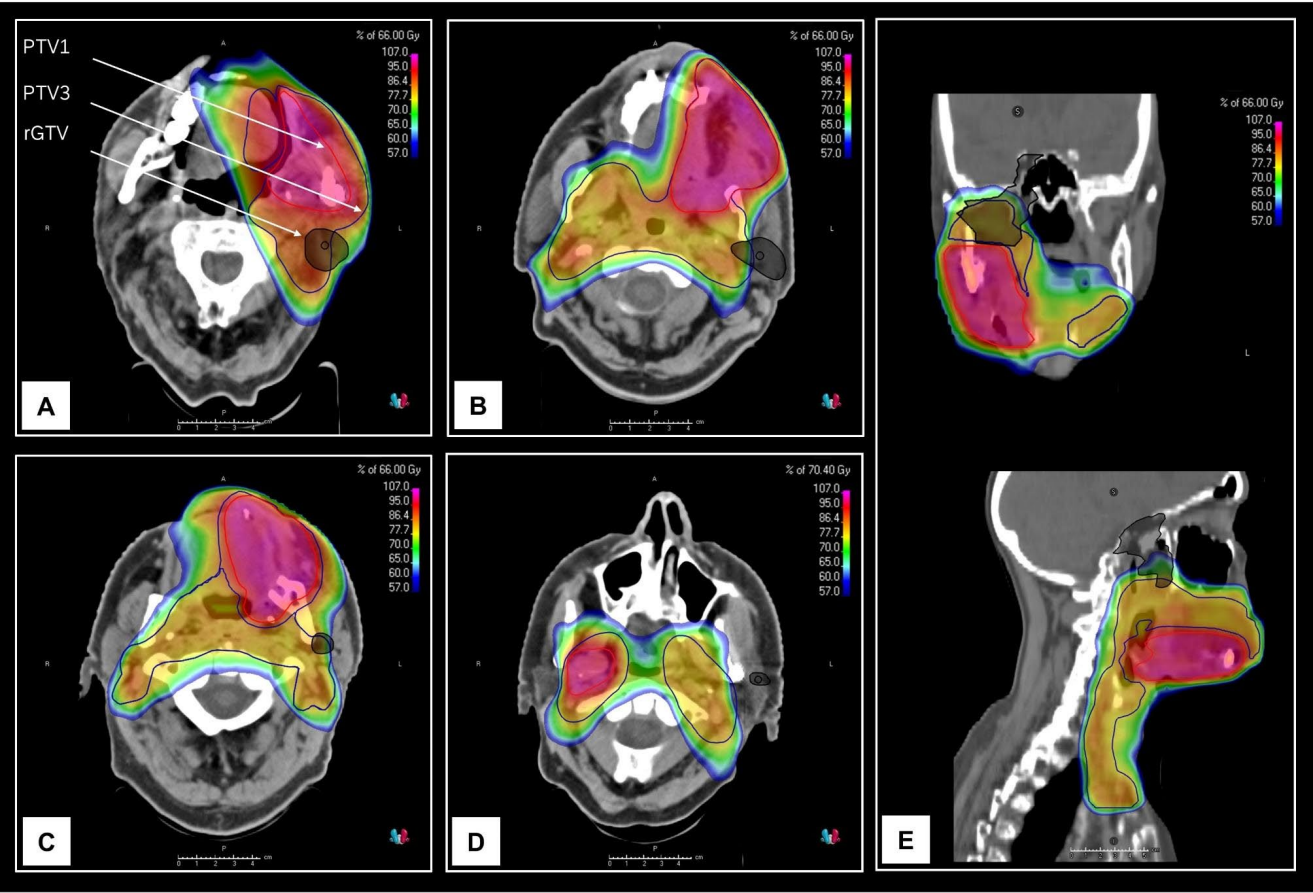
Examples of retrograde lymphatic spread into ipsilateral parotid lymph nodes in 4 patients with primary tumor resection, ipsilateral or bilateral ND and in all cases ECE + ipsilateral lymph node metastases and R1 or close margin of the primary tumor (**A**-**D**). (**A**) SCC of the left lower alveolar ridge, pT4a pN3b ECE + close margin Pn0 LV0 G2, ND level I-III ipsilateral, recurrence type D intermediate dose. (**B**) SCC left buccal mucosa, pT4b pN3b ECE + R1 Pn0 LV1 G3, ND level I-III bilateral, recurrence type E. (**C**) SCC left maxilla, pT4a pN3b ECE + R1 Pn0 LV1 G2, ND level I-III ipsilateral, recurrence type D low dose. (**D**) SCC left anterior floor of mouth, pT4a pN3b ECE + R1 Pn1 LV1 G2, ND level I-III bilateral, recurrence type E. (**E**) Example of retrograde perineural spread along the mandibular nerve through the base of skull after resection of a SCC of the right lower alveolar ridge, pT4a pN1 ECE- R1 Pn1 LV0 G2, recurrence type D low dose. ND = neck dissection, SCC = squamous cell carcinoma, ECE = extracapsular extension

The 2 patients with type D low-dose failure both experienced cranial dispersion as recurrence growth pattern. One had a resected carcinoma of the right lower alveolar ridge with perineural invasion. Recurrence occurred within the right masticator space, infiltrating through the right foramen ovale into the osseous base of the skull, presumably as perineural spread along the mandibular nerve (Fig. 3E). The other had a resected carcinoma of the lower left alveolar ridge with recurrence at the flap interface, extending along the left ramus mandibulae into the left masticator space.

Of the 5 patients with type E extraneous dose failures, 3 were classified as secondary cancers. Extensive outgrowth of recurrent disease from the high-dose and low-dose target volumes into cranial out of field areas was the reason for the remaining 2 type E failures.

### Cases of regional recurrence

Of 12 analyzable patients with regional recurrence only and 2 patients with regional recurrence and concomitant distant failure, 4 (29%) had high-dose failures (2 type A and 2 type B). Of 10 (71%) non high-dose failures 1 was classified as type C intermediate-dose, 1 as type D intermediate-dose, 6 as type C low-dose and 2 as type E. Focusing on non-central high-dose failures: One type B peripheral high-dose failure occurred in a patient with resected left maxilla carcinoma, flap and plate reconstruction, ipsilateral neck dissection, and risk factors like R1 margin status, extracapsular extension and lymphovascular invasion. The rGTV originated in the left retromandibular region, extending into the parotid space with a second recurrence within the parotid (type D, Fig. 3C). This regional therapy failure, indicative of retrograde lymphatic spread into the (partially) RT-spared parotid, was seen in 5/40 patients. Figures 3A-D display 4 cases. These patients shared common features such as extensive reconstructive surgery and ipsilateral lymph node metastases with ECE + and lymphovascular invasion in 4/5 cases.

**Fig. 4:**
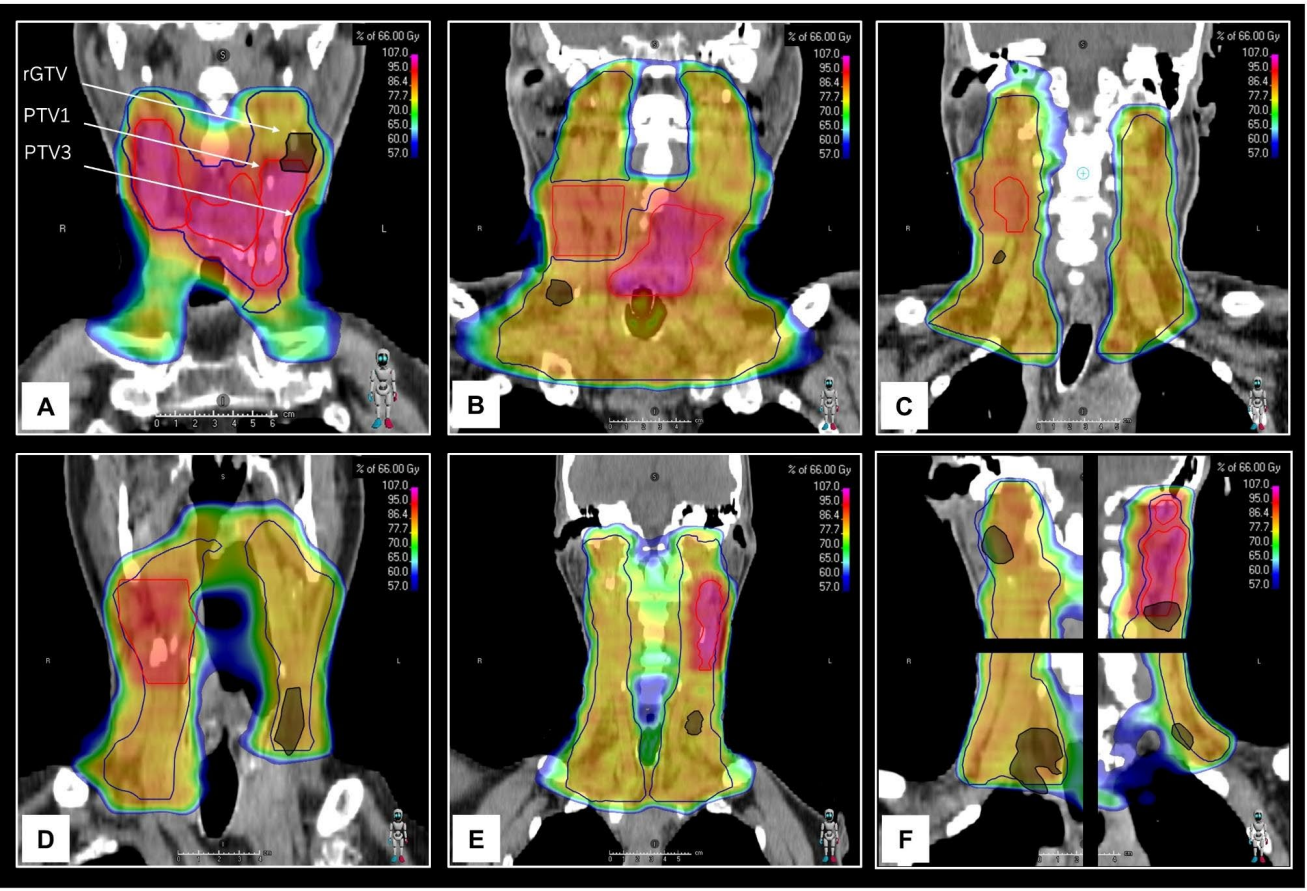
Six cases of type C low dose recurrence within the elective neck volume (**A**-**F**). (**A**) SCC of the hypopharynx, initial cN+/pN + level II right and left, rcN+/rpN + level II right (not shown) and level II left. (**B**) SCC of the larynx, initial pN + level II-V right, rcN+/rpN + level IV right. (**C**) SCC of the lower right alveolar ridge, initial cN+/pN + level I-II right, rcN + level III right. (**D**) SCC of the left boarder of the tongue, initial cN+/pN + level II right, rcN+/rpN + level III left. (**E**) SCC of the left boarder of the tongue, initial cN+/pN + level II left, rcN + level IV left and concomitant distant metastases. (**F**) SCC of the left boarder of the tongue, initial cN + level II right with pN0, cN+/pN + level II left, rcN + level II and IV right, III and V left and concomitant distant metastases. SCC = squamous cell carcinoma

The 6 patients with regional type C low-dose failures in electively treated neck volumes, all had bilateral neck dissection and bilateral neck irradiation. In 4/6 cases recurrences occurred solely within the neck side of initial lymph node involvement (Fig. 4). Two of the 6 patients with regional failures in elective volumes already had advanced disease recurrence with concomitant distant failure and subcutaneous metastases.

### Cases of locoregional recurrences

Patients with locoregional recurrence, by definition, had multifocal recurrence involving the primary tumor site and regional areas. Of 3 patients with locoregional recurrence and 2 patients with locoregional and concomitant distant failure 4 (80%) had high-dose failures: 3 were classified as type A (dominant type), with concomitant type B or type E failures in cranial or upper cervical lymph node areas.

## Discussion

We evaluated a mixed-case cohort of HNSCC patients treated with curative intent PO-IMRT. Three-year control rates for HPV-negative patients were 84.0% locally, 87.0% regionally, and 87.0% for distant sites, aligning with findings from other studies [18–22]. The Kaplan-Meier estimates for post-recurrence median OS in OCSCC patients were 12.7 months for local/regional recurrences and 3.2 months for distant metastases, which compared favorably with other reports [23].

Various analytical methods have been developed to classify recurrence volumes in order to estimate their etiology. The volume-based method, which quantifies the overlap of recurrence and target volumes [20, 24–26], or the 95% isodose [19, 27–30], often results in a marginal classification bias, especially in cases of delayed diagnosis and larger recurrence volumes. Furthermore, it does not incorporate dosimetry when based solely on target volumes [1, 3, 31]. The point-based or focal method, which determines the spatial relationship of the recurrence volume’s center of mass to the initial target volume, offers more accuracy in the definitive treatment setting [31, 32], but is limited by its assumption of homogeneous concentric tumor growth, often not applicable post-surgery due to anatomical changes.

Addressing these shortcomings, a combined spatial and dosimetric analysis method has been introduced by Mohammed et al. [2, 4]. This approach utilizes deformable image registration with superior accuracy and reproducibility compared to rigid image registration [2, 31] and introduces a more etiology-indicative failure type classification.

In our cohort of 203 HNSCC patients, we observed 56 recurrences. Excluding patients with distant metastasis only and 2 patients without recurrence imaging left 40 patients for analysis. Of those, 25% were classified as type B or D, conventionally classified as “marginal” recurrences. However, a detailed case review revealed that 80% of these recurrences were due to tumor outgrowth from high to low-dose target volumes, rather than incorrect delineation. Therefore, while the combined method provides a detailed classification, an accurate assessment of recurrence etiology should always include a clinically informed case review.

Together, we observed 75% in-field, 5% marginal, and 20% out-of-field recurrences, with the latter including 50% secondary cancers, indicating effective target volume definition at our institution.

Furthermore, 48% of the cases were high-dose failures, suggesting tumor resistance to PO-IMRT. Strategies to prevent such failures might include the optimization of head and neck surgical techniques to increase the rate of R0 resections [33]. Moreover, it underlines the necessity to develop novel predictive markers for RT response, thereby refining postoperative risk stratification and guiding treatment intensification.

Particularly notable was a failure pattern involving retrograde lymphatic spread to the ipsilateral parotid area observed in 5/40 patients, with parotid-sparing IMRT in 3/5 cases. This failure pattern has also been described by previous series [4, 26]. We suggest reevaluation of parotid-sparing strategies to balance reduced radiation-induced xerostomia with improved locoregional control in patients who fulfill several recurrence risk factors.

Another key observation was the retrograde perineural spread along cranial nerves, resulting in extensive recurrences, as reported by other series [4, 34, 35]. This emphasizes the importance of covering nerve paths near primary sites in radiation planning, especially for tumors with a high potential for cranial dispersion, such as advanced tumors located in the buccal mucosa, retromolar trigone, and alveolar ridges. Routes of spread along V2, V3, or directly through the temporalis muscle as well as the related communicating extracranial spaces have been described in depth by Lin et al. [29].

To reduce toxicity, several trials are exploring the safety of decreasing radiation volumes to neck areas with an empirically low risk for nodal micrometastases [5–7, 36, 37]. Currently, these areas are treated electively with doses up to 54 Gy. This approach is particularly relevant for OCSCC patients, who often undergo extensive bilateral elective neck irradiation postoperatively, increasing the risk of oral complications and dysphagia [38]. Nonetheless, de-escalation poses challenges to locoregional control, especially in this prognostically unfavorable HPV-negative HNSCC subgroup. Therefore, updated recurrence analyses providing dose-level-specific recurrence probabilities could guide such volume modulation trials.

Unilateral neck irradiation has shown favorable toxicity outcomes in OCSCC patients [7, 39, 40]. However, European consensus recommends this approach only for lateralized OCSCC with limited nodal involvement [12], to mitigate the risk of contralateral lymphatic spread, particularly in centrally located tumors [4, 18, 19, 41], with extensive nodal involvement or deep primary tumor invasion [12, 39, 42].

Notably, Contreras et al. reported a 97% control rate in the unirradiated neck when omitting elective irradiation of pN0 neck sides in 72 HNSCC patients, despite 71% had tumors involving midline structures. In this trial most patients received bilateral neck dissection and preoperative 18FDG-PET/CT staging [7].

Our analysis showed that 27.5% (11/40) of local/regional recurrences occurred in low-risk regions (type C/D), but true nodal failures were only 15% (6/40), indicating an overall isolated regional recurrence risk of 3% (6/203) in electively treated neck volumes. This aligns with other studies reporting failure rates of 1–10% in corresponding low-risk volumes [4, 18, 20, 21, 23] (median 3%, Supplementary Table 2). Therefore, stronger risk adaptation of electively treated neck volumes could be viable for HPV-negative HNSCC, especially in OCSCC, provided comprehensive preoperative staging is conducted. Despite its retrospective nature and potential biases from being conducted at a high-volume center, this study represents one of the largest mixed-case analyses of failure patterns in PO-IMRT in HNSCC, underscoring the importance of tailored risk adaptation in treatment planning to balance efficacy and toxicity.

## Conclusion

In this mixed-case, predominantly HPV-negative HNSCC cohort, the most common failure type following postoperative IMRT occurred centrally or marginally to high-risk/high-dose volumes, indicating primary tumor resistance as the key driver of therapy failure. Given the lack of benefit from unguided dose escalation, this underscores the need to refine current postoperative HNSCC risk stratification towards a tumor biology-based dose prescription. Among 203 patients analyzed, we noted 6 regional failures in electively treated neck regions. To reduce treatment-related toxicity, volumetric de-escalation of these regions could be viable not only for HPV-positive, but also HPV-negative HNSCC. This approach requires comprehensive preoperative diagnostics and precise nodal staging, with further validation in prospective trials.

## Electronic supplementary material

Below is the link to the electronic supplementary material.

Supplementary Material 1

## Data Availability

No datasets were generated or analysed during the current study.

## Abbreviations

3D-CRT: Three-dimension conformal radiotherapy
CTV: Clinical target volume
CT: Computed tomography
CTCAE: Common Terminology Criteria for Adverse Events
DAHANCA: Danish Head and Neck Cancer Group
DIR: Deformable image registration
DM: Distant metastasis
ECE: Extracapsular extension
HIRO: Heidelberg Institute for Radiation Oncology
HNSCC: Head and neck squamous cell carcinoma
HPV: Human papillomavirus
HPSCC: Squamous cell carcinoma of the hypopharynx
IMRT: Intensit-modulated radiotherapy
LR: Local recurrence
LSCC: Squamous cell carcinoma of the larynx
mos.: Months
MRI: Magnetic resonance imaging
ND: Neck dissection
OCSCC: Squamous cell carcinoma of the oral cavity
OPSCC: Squamous cell carcinoma of the oropharynx
pCT: Planning computed tomography
PO-IMRT: Postoperative intensity-modulated radiotherapy
PORT: Postoperative radiotherapy
PTV: Planning target volume
rCT: Computed tomography documenting recurrence
rGTV: Recurrent gross tumor volume
rMRI: Magnetic resonance imaging documenting recurrence
RR: Regional recurrence
RT: Radiotherapy
TTP: Total treatment package time
