# Supplementary material for: Combined Spatial and Dosimetric Recurrence Pattern Analysis in Head and Neck Squamous Cell Carcinoma Following Postoperative (Chemo)radiotherapy
Source: Radiat Oncol. 2025 Apr 23;20:63. doi: 10.1186/s13014-025-02641-8 (PMC12020044; doi:10.1186/s13014-025-02641-8)
Supplement: Supplementary file 1 — Supplementary Material 1 [file 13014_2025_2641_MOESM1_ESM.docx]

Supplementary Appendix:

Combined spatial and dosimetric recurrence pattern analysis in head and neck squamous cell carcinoma patients following postoperative (chemo)radiotherapy

Philipp Schröter^a–e^**^*^**, Hoi Hin Lau^a–e^, Florian Stritzke^a–e^, Henrik Franke^a–e^, Katharina Weusthof^a–e^, Sebastian Regnery^a–e^, Lukas Bauer^a–e^, Maximilian Deng^a–e^, Katharina Dvornikovich^a–e^, Anna Hofmann^a–e^, Lars Wessel^a–e^, Karl Semmelmayer^g^, Julius Moratin^g^, Oliver Ristow^g^, Jürgen Hoffmann^g^, Peter K Plinkert^h^, Gerhard Dyckhoff^h^, Jürgen Debus^a-f^, Thomas Held^a-f^

1. Heidelberg University Hospital, Department of Radiation Oncology, Heidelberg, Germany
2. Heidelberg Institute of Radiation Oncology (HIRO), Heidelberg, Germany
3. National Center for Tumor diseases (NCT), Heidelberg, Germany
4. Clinical Cooperation Unit Radiation Oncology, German Cancer Research Center (DKFZ), Heidelberg, Germany
5. Heidelberg Ion Beam Therapy Center (HIT), Heidelberg, Germany
6. German Cancer Consortium (DKTK), partner site Heidelberg, German Cancer Research Center (DKFZ), Heidelberg, Germany
7. Heidelberg University Hospital, Department of Oral and Cranio-Maxillofacial Surgery, Heidelberg, Germany
8. Heidelberg University Hospital, Department of Otorhinolaryngology, Head and Neck Surgery, Heidelberg, Germany


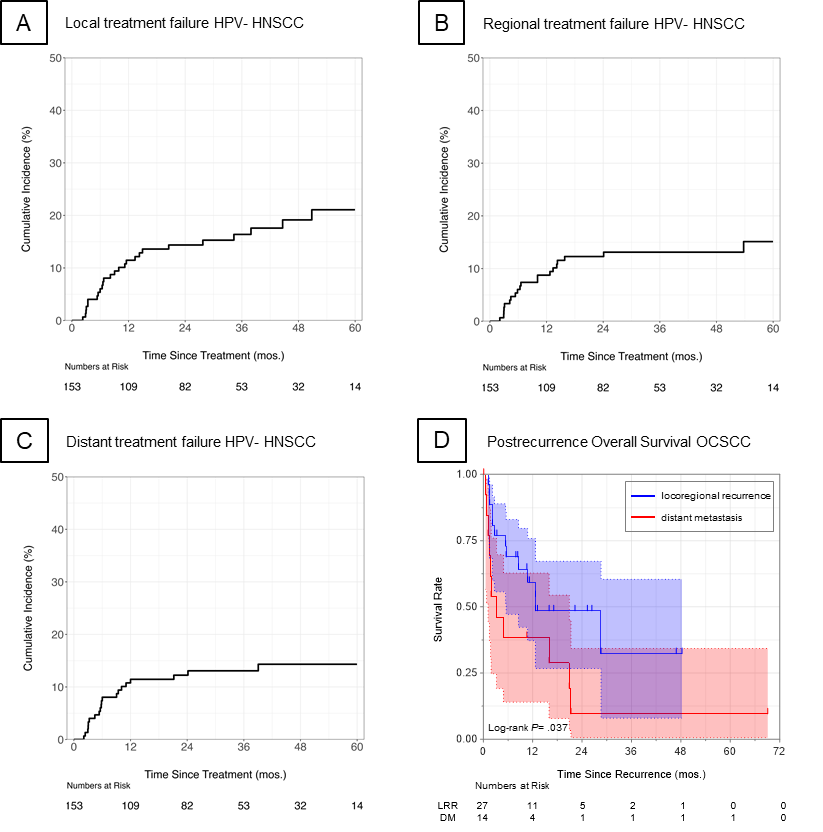


**Supplemantal Figure 1.** (A-C) Cumulative incidence of local, regional and distant treatment failure in HPV- HNSCC patients, taking competing events into account. (D) Kaplan-Meier estimate of postrecurrence overall survival in patients with oral cavity squamous cell carcinoma with local, regional or locoregional recurrence vs. distant metastasis. HPV = human papillomavirus, HNSCC = head and neck squamous cell carcinoma, OCSCC = oral cavity squamous cell carcinoma, LRR = locoregional recurrence, DM = distant metastasis, mos.= months

**Supplemental Table 1**

Details of analysis of patients with local, regional or locoregional recurrence ± distant metastasis (n=42)

| Patient | Primary Tumor Site | pTNM  (8th edition) | Surgical  Margins | ECE | DOI | PNI | LVI | ND  Laterality | Neck RT  Laterality | CTx | PTV1  Dose (Gy/n. of Fractions) | Local/Regional/Locoregional/  Distant | Recurrence  Classification  and related  PTV | Recurrence  Site | Potential Etiology |
| --- | --- | --- | --- | --- | --- | --- | --- | --- | --- | --- | --- | --- | --- | --- | --- |
| 1 | oral cavity  tongue left | T3 N2b (2/12) | CM | neg | >10mm | 1 | 1 | bilateral | bilateral | none | 66/30 | regional | B 60  E | level Ib left,  level Ib right | resistance  delineation |
| 2 | oral cavity  tongue left | T3 N1 (1/17) | R0 | neg | >10mm | 0 | 1 | bilateral | bilateral | none | 66/30 | regional | C 57 | level III left | resistance |
| 3 | oral cavity  tongue left | T1 N2c (2/46) | CM | neg | x | 0 | 0 | bilateral | bilateral | none | 60/30 | regional | C 54 | level III left | risk assessment ENI |
| 4 | oral cavity  tongue left | T1b N3b (4/35) | R0 | pos | ≤5mm | 0 | 1 | bilateral | bilateral | Cisplatin | 66/30 | regional/  distant | C 54  D 54  D 54  D 54  E  E  E | level III left  level Vb left  level IIa right  level Iva right  subcutaneous  subcutaneous  subcutaneous | risk assessment ENI  risk assessment ENI  risk assessment ENI  risk assessment ENI  aggressive failure  aggressive failure  aggressive failure |
| 5 | oral cavity  tongue right | T3 N0 (0/18) | CM |  | >10mm | 1 | 0 | bilateral | bilateral | none | 66/30 | local | C 54 | tongue left | secondary cancer |
| 6 | oral cavity  tongue right | T2 N2b (4/62) | R0 | neg | x | 0 | 0 | bilateral | bilateral | none | 60/30 | local/  distant | C 54 | primary | outgrowth/resistance |
| 7 | oral cavity  tongue left | T3 N1 (1/9) | CM | neg | >10mm | 1 | 1 | bilateral | bilateral | none | 60/30 | local | E | FOM right | secondary cancer |
| 8 | oral cavity  tongue left | T4a N3b (6/31) | CM | pos | x | 1 | 1 | bilateral | bilateral | Cisplatin | 66/30 | local/  distant | B 66 | primary | outgrowth/resistance |
| 9 | oral cavity  tongue right | T2 N0 (0/29) | CM |  | >5mm, ≤10mm | 1 | 1 | bilateral | bilateral | none | 60/30 | local | C 54 | primary | outgrowth/resistance |
| 10 | oral cavity  tongue left | T2 N2b (5/44) | CM | neg | >5mm, ≤10mm | 1 | 1 | bilateral | bilateral | none | 66/30 | regional/  distant | C 54  E  E  E  E | level IVa left  cutaneous  cutaneous  cutaneous  cutaneous | risk assessment ENI  aggressive failure  aggressive failure  aggressive failure  aggressive failure |
| 11 | oral cavity  tongue right | T3 cN1 | CM |  | x | 0 | 0 | none | bilateral | Cisplatin | 70.4/32 | regional | A 70.4 | level IIa right | resistance |
| 12 | oral cavity buccal mucosa left | T4b N3b (5/16) | R1 | pos | >10mm | 0 | 1 | ipsilateral | bilateral | Cisplatin | 66/30 | regional | E | left parotid | retrograde spread |
| 13 | oral cavity  FOM left | T4a N0 (0/46) | CM |  | >10mm | 1 | 0 | bilateral | bilateral | none | 60/30 | local | E | primary  cranial dispersion | outgrowth/resistance |
| 14 | oral cavity  FOM anterior | T1 N3b (2/24) | CM | pos | ≤5mm | x | 1 | bilateral | bilateral | Carbo-platin | 66/30 | local | E | larynx | secondary cancer |
| 15 | oral cavity  FOM left | T2 N1 (1/35) | R1 | neg | >5mm, ≤10mm | 1 | 1 | bilateral | bilateral | none | 66/30 | local/  distant | B 66 | primary | outgrowth/resistance |

**Supplemental Table 1** (continued)

| Patient | Primary Tumor Site | pTNM  (8th edition) | Surgical  Margins | ECE | DOI | PNI | LVI | ND  Laterality | Neck RT  Laterality | CTx | PTV1  Dose (Gy/n. of Fractions) | Local/Regional/Locoregional/  Distant | Recurrence  Classification  and related  PTV | Recurrence  Site | Potential Etiology |
| --- | --- | --- | --- | --- | --- | --- | --- | --- | --- | --- | --- | --- | --- | --- | --- |
| 16 | oral cavity  FOM anterior/left | T4a N3b (9/25) | R1 | pos | >10mm | 1 | 1 | bilateral | bilateral | none | 70.4/32 | regional | E  E  E | left parotid  cutaneous  cutaneous | retrograde spread  aggressive failure  aggressive failure |
| 17 | oral cavity  FOM  anterior | T4a N3b (4/31) | R1 | pos | x | x | 1 | bilateral | bilateral | Cisplatin | 70.4/32 | locoregional/  distant | A 70.4  E  E | primary  masseter left parotid left | resistance  outgrowth  retrograde spread |
| 18 | oral cavity  lower left alveolar  process and gingiva | T4a N2b (5/32) | CM | neg | >10mm | 1 | 1 | bilateral | bilateral | none | 60/30 | locoregional | A 60  E  E | primary  primary  subcutaneous | resistance  delineation  aggressive failure |
| 19 | oral cavity  lower right alveolar  process and gingiva | T4a N0 (0/21) | R0 |  | >5mm, ≤10mm | 0 | 0 | bilateral | bilateral | none | 60/30 | local | E | palate | secondary cancer |
| 20 | oral cavity  lower left alveolar  process and gingiva | T4a N0 (0/15) | CM |  | >10mm | 0 | 0 | bilateral | bilateral | none | 60/30 | local | D 54 | primary  cranial dispersion | outgrowth/resistance |
| 21 | oral cavity  lower left alveolar  process and gingiva | T4a N0 (0/38) | R0 |  | >10mm | 0 | 0 | bilateral | bilateral | none | 60/30 | locoregional | B 60  E  E  E | primary  primary  subcutaneous  subcutaneous | outgrowth/resistance outgrowth/resistance  aggressive failure  aggressive failure |
| 22 | oral cavity  lower right alveolar  process and gingiva | T4a N3b (4/21) | x | pos | x | 0 | 1 | bilateral | bilateral | none | 72.6/33 | local | A 72.6 | primary | resistance |
| 23 | oral cavity  lower left alveolar process and gingiva | T4a N3b (2/13) | CM | pos | >5mm, ≤10mm | 0 | 0 | ipsilateral | ipsilateral | none | 66/30 | regional | D 57 | left parotid | retrograde spread |
| 24 | oral cavity  upper left alveolar  process and gingiva | T4a N3b (2/14) | R1 | pos | x | 0 | 1 | ipsilateral | bilateral | Cisplatin | 70.5/30 | regional | B 70.5  D 54 | Retro-mandibular  left parotid | outgrowth/resistance  retrograde spread |
| 25 | oral cavity  lower left alveolar  process and gingiva | T4a N0 (0/15) | R0 |  | ≤5mm | x | 0 | ipsilateral | bilateral | none | 66/30 | local | E | primary  cranial dispersion | outgrowth/resistance |
| 26 | oral cavity  upper left alveolar  process and gingiva | T4a N0 (0/11) | R1 |  | >10mm | x | 0 | ipsilateral | bilateral | Cisplatin | 66/30 | local | A 66  A 66  E | primary  primary  nasopharynx | resistance  resistance  multilevel failure |
| 27 | oral cavity  mandible right | T4a N0 (0/21) | CM |  | >5mm, ≤10mm | 0 | 0 | ipsilateral | bilateral | none | 66/30 | local | A 66 | primary | resistance |
| 28 | oral cavity  lower right  alveolar process and gingiva | T4a N3b (3/50) | R1 | pos | >10mm | 1 | 1 | bilateral | bilateral | Carbo-platin | 66/30 | Local/  distant | A 66 | primary | resistance |

Recurrence Classification and related PTV: in case of multiple recurrence volumes, the highest classification was considered dominant, Potential Etiology: suggested cause of recurrence by consensus reading of clinicopathological tumor information, radiotherapy plan and appearance of recurrence volume on recurrence CT/MRI, primary: indicates that the recurrence volume originated from the primary (pre-operative) tumor region. FOM = floor of mouth, ECE = extracapsular extension, ENI = Elective Neck Irradiation (CTV3/PTV54Gy), CM = close margin (1-5mm), PNI = perineural invasion, LVI = lymphovascular invasion, CTx = chemotherapy, PTV = planning target volume, *for these patients no recurrence imaging was available, but clinical information was provided

**Supplemental Table 1** (continued)

| Patient | Primary Tumor Site | pTNM  (8th edition) | Surgical  Margins | ECE | DOI | PNI | LVI | ND  Laterality | Neck RT  Laterality | CTx | PTV1  Dose (Gy/n. of Fractions) | Local/Regional/Locoregional/  Distant | Recurrence  Classification  and related  PTV | Recurrence  Site | Potential Etiology |
| --- | --- | --- | --- | --- | --- | --- | --- | --- | --- | --- | --- | --- | --- | --- | --- |
| 29 | oral cavity  lower left alveolar  process and gingiva | T4a N0 (0/18) | CM |  | >5mm, ≤10mm | 0 | 0 | ipsilateral | none | none | 60/30 | local | A 60 | primary | resistance |
| 30 | oral cavity  lower left alveolar  process and gingiva left | T4a N0 (0/30) | R1 |  | x | 1 | 1 | bilateral | bilateral | Cisplatin | 70.4/32 | local  local | B 70.4  B 70.4 | primary  primary | outgrowth/resistance outgrowth/resistance |
| 31 | oral cavity  lower left/right alveolar process and gingiva | T4a N0 (0/26) | x |  | >10mm | 1 | 0 | bilateral | none | none | 66/33 | local | B 66 | primary | outgrowth/resistance/ delineation |
| 32 | oral cavity  lower right alveolar  process and gingiva | T4a N1 (1/52) | R1 | neg | >5mm, ≤10mm | 1 | 0 | bilateral | bilateral | Cisplatin | 66/30 | local/  distant | D 54 | primary  cranial dispersion | outgrowth/resistance |
| 33 | oral cavity  lower right alveolar  process and gingiva right | T4a N1 (1/73) | R1 | neg | >10mm | 1 | 0 | bilateral | bilateral | none | 60/30 | regional | C 54 | level III right | risk assessment ENI |
| 34 | Larynx  transglottic | T4a N3b (3/42) | CM | pos | >10mm | 1 | 1 | bilateral | bilateral | Carbo-platin | 66/30 | regional | A 66  A 66 | level III right  level IVa right | resistance  resistance |
| 35 | hypopharynx | T3 N3b (1/13) | R0 | neg | >10mm | 1 | 1 | bilateral | bilateral | Cetuxi-mab | 66/30 | locoregional/  distant | A 66  B 66 | level III left  primary | resistance  outgrowth/resistance |
| 36 | larynx, supraglottic | T3 N3b (7/27) | CM | pos | x | 0 | 1 | bilateral | bilateral | Cisplatin | 70.4/32 | locoregional | E  D 57  D 57 | nasopharynx  level IIa left  level IIb left | secondary cancer  secondary cancer  secondary cancer |
| 37 | hypopharynx | T3 N3b (3/78) | R1 | pos | ≤5mm | 1 | 1 | bilateral | bilateral | Cisplatin | 66/30 | regional | C 54  C 54 | level IIa left  level IIb right | risk assessment ENI  risk assessment ENI |
| 38 | larynx  glottic | T4a N3b (3/33) | R0 | pos | x | x | 1 | bilateral | bilateral | Cisplatin | 66/30 | regional | C 54 | level IVa right | risk assessment ENI |
| 39 | larynx  supraglottic | T3 N2a (1/32) | R0 | pos | x | 0 | 0 | bilateral | bilateral | Cisplatin | 70.4/32 | local | A 70.4 | primary | resistance |
| 40 | Oropharynx | T3 N2b (2/13) | R0 | neg | >10mm | 0 | 1 | bilateral | bilateral | none | 66/33 | local | A 66 | primary | resistance |
| 41* | oral cavity  tongue right | T3 N1 (2/24) | R0 | neg | >10mm | 1 | 1 | bilateral | bilateral | none | 66/30 | regional | - | - | - |
| 42* | oral cavity, alveolar process and gingiva right | T4 N0 (0/28) | close margin |  | x | 0 | 0 | bilateral | ipsilateral | none | 66/30 | local | - | - | - |

**Supplemental Table 2**

Summary of retrospective recurrence pattern analyses

| Author, Year, Institution,  Country | Time Period,  Median f/u | No. of Patients,  Treatment | Sites | ND.  Absolut,  Bilateral | Median Time  to LRF | LRF  LRF + DM | Isolated LF  Isolated RF  Synchron. LRF | LRF in High-/Intermed.-  Dose-Field^a^ | LRF in Elective-  Dose-Field^b^ | LRF out of Field^c^ |
| --- | --- | --- | --- | --- | --- | --- | --- | --- | --- | --- |
| Waldram 2020  Leeds CC, UK* | 2013 – 2016  36 mos. | 101  IMRT | OCSCC | 96% (97/101)  27% (27/101) | 6 mos. | 19% (19)  3% (3) | 8% (8)  10% (10)  1% (1) | 14% (14/101)^d^  64% (14/22) | 4% (4/101)^e^  18% (4/22) | 4% (4/101) |
| Mohamed 2017  MDACC, US | 2000 – 2012  35 mos. | 289  IMRT | OCSCC | 93% (268/289)  24% (64/268) | 4 mos. | 19% (54)^f^  NA | 9% (26)  7% (19)  3% (9) | 14% (40/280)^g^  72% (40/54) | 1,8% (5/280)^h^  13% (4/54) | 3% (9/280) |
| Leeman 2017  MSKCC, US* | 2001 – 2013  65 mos. | 125  IMRT | OCSCC | NA  NA | NA | 23% (29)  4% (5) | NA  12% (15)  NA | NA | 9.6% (12/125)^i^  27% (12/45) | 0% |
| Ooishi 2016  NCC Hosp. East  Kashiwanoha; JP* | 2006 – 2013  54 mos. | 122  IMRT | OCSCC (59)  HPSCC (31)  OPSCC (14)  LSCC (10)  CUP (8) | NA | 8 mos. | 20% (24)  7% (8) | NA  NA  NA | 23% (28/122)^j^  88% (28/32) | 3% (4/122)^k^  13% (4/32) | 2% (3/122) |
| Chakraborty 2015  Malabar CC, IN | 2012 – 2014  24 mos. | 75  IMRT | OCSCC | 100% (75/75)  21% (16/75) | 7 mos. | 11% (8)  NA | 1% (1)  9% (7)  NA | 4% (3/75)^l^  38% (3/8) | 3% (2/75)^m^  25% (2/8) | 4% (3/75) |
| Ferreira, 2015  IPO Coimbra, PT | 2007 – 2013  NA | 151  IMRT  3D-CRT | OCSCC  OPSCC  HPSCC  LSCC | NA | NA | 5% (8/151)  NA | 1% (4)  1% (1)  2% (3) | NA | 1-2% (1-3/151)^n^ | 1-4% (1-4/151) |
| Stromberger 2014*  Charité Berlin, DE | 2001 – 2010  41 mos. | 129  IMRT | OCSCC (19)  OPSCC (96)  HPSCC (8)  LSCC (4)  Parotid (2) | NA | 9 mos.  (local)  17 mos. (regional)  19 mos. (distant) | 9% (11)  3% (4) | 4% (5)  2% (3)  2% (3) | 6% (7/124)^o^  70% (7/10) | 1% (1/124)^p^  10% (1/10) | 2% (2/124) |
| Chen 2011  UC Davis CCC, US* | 2003 – 2009  27 mos. | 90  IMRT | OCSCC (43)  OPSCC (20)  HPSCC (12)  LSCC (15) | 94% (85/90)  27% (23/85) | 12 mos. | 11% (10)  8% (7) | NA  NA  NA | 12% (11/90)^q^  71% (11/17) | 7% (6/90)^r^  35% (6/17) | 0% (0/90) |

a: LRF in high-/intermediate-dose-field was defined as in-field- or marginal-recurrence in a high-/intermediate-dose-field b: LRF in elective-dose-field was defined as any in-field- or marginal-recurrence in a low-dose-field c: LRF out of field as defined as „extraneous recurrence“ d: 10 central high-dose-field, 4 peripheral high-dose-field (CTV60-66Gy in 30/33 Fx) e: 2 central elective-dose-filed, 2 peripheral elective-dose-field (CTV54-56Gy in 30/33 Fx) f: 54 of 63 recurrences where analyzed, exclusion of 4 patients with post-surgical recurrence prior to initiation of IMRT, 3 with no retrievable IMRT plans, 2 with no available imaging documenting recurrence g: 30 central high-dose-field, 2 peripheral high-dose-field (CTV60-66Gy), 6 central intermediate-dose-field, 2 peripheral intermediate-dose-field (CTV57Gy) h: 4 isolated regional recurrences in central low-dose-field (CTV54Gy), 1 low neck failure in low-dose-field i: 15 patients with isolated nodal recurrences in elective target volumes, 15/15 patients had unilateral neck dissection only, nodal recurrence occurred in dissected regions in 3 patients, contralateral (undissected regions) in 9 patients and both ipsilateral and contralateral in 3 patients j: 28 in-field- and/or marginal-recurrence in the high-/intermediate-dose-field (CTV60-66Gy in 33 Fx) k: 4 recurrences that occurred „out-of-field within the low-risk CTV“ (CTV54Gy in 33 Fx) l: 3 high-dose-field (CTV60-66Gy) m: 2 low-dose-field (CTV54-56Gy) n: 3 recurrence volumes that occurred in-field or marginal to low-risk CTV (CTV50-54Gy), 3 volumes corresponds to 1-3 patients o: 10 of 15 recurrences were analyzed for dosimetry, exclusion of 5 patients with no adequate imaging, 7 in-field-recurrences in the high-dose-field (CTV1 = 56Gy in intermediate-risk-patients and CTV1 = 64Gy in high-risk-patients, low-dose-field = CTV2 = 50-54Gy in this analysis) p: 1 recurrence as a marginal recurrence to CTV1 or CTV2, interpreted as a potential recurrence in the low-dose-field q: 4 recurrences at primary site and 4 in high-risk cervical neck (CTV60-66Gy), 2 marginal recurrences to CTV60Gy, 1 marginal recurrence to CTV56Gy r: 3 recurrences in the supraclavicular neck (CTV50-54Gy), 3 marginal to CTV54Gy * Patients with distant metastases were taken into account for failure analysis of LRF.

f/u = follow-up, ND = neck dissection, LRF = locoregional failure, DM = distant metastasis, OCSCC = oral cavity squamous cell carcinoma, OPSCC = oropharyngeal squamous cell carcinoma, HPSCC = hypopharyngeal squamous cell carcinoma, LSCC = laryngeal squamous cell carcinoma, CUP = cancer of unknown primary, mos.= month, NA = not available, Fx = fraction
